# *Actinomyces europaeus* Isolated from a Breast Abscess in a Penicillin-Allergic Patient

**DOI:** 10.1155/2018/6708614

**Published:** 2018-06-20

**Authors:** Sarah E. White, Stephen D. Woolley

**Affiliations:** ^1^Foundation Programme, Royal Liverpool University Hospital, Liverpool, UK; ^2^Medical Microbiology Department, Liverpool Clinical Laboratories, Royal Liverpool University Hospital, Liverpool, UK; ^3^Clinical Sciences, Liverpool School of Tropical Medicine, Liverpool, UK

## Abstract

This is a case of *Actinomyces europaeus* in the breast abscess of a penicillin-allergic woman. The mainstay of treatment for actinomycosis is penicillin, and there is a lack of literature describing nonpenicillin treatment options. A 69-year-old woman presented acutely with a breast abscess which was managed with incision and drainage and antibiotic therapy to good response. 21 days after presentation, *Actinomyces* were grown from the culture of pus, so the patient was recalled and more rigorous treatment and follow-up were initiated. The penicillin allergy led to difficulty in the identification of an appropriate antimicrobial agent that was also logistically feasible to be given on an outpatient IV basis. IV tigecycline followed by oral clarithromycin was found to be effective treatment.

## 1. Introduction

*Actinomyces* species are normal commensal bacteria that can be found in the oral cavity and the lower reproductive tract of women [1]. The bacteria are Gram-positive, filamentous, non-acid-fast, and anaerobic-to-microaerophilic [2]. This species is pathogenic but nonvirulent, so infection is often opportunistic leading to actinomycosis which is a rare subacute-to-chronic infection [3]. Actinomycosis is characterised by localised spread via abscess and sinus tracts with granulomatous inflammation [2]. Due to this propensity for local spread, identified infections should be investigated for deep sources which may contribute to an ongoing insidious clinical source or may be mistaken for other pathologies such as carcinomas [4]. Diagnosis of actinomycosis is mainly by examination of microbiological samples because clinical suspicion is low and clinicians are unfamiliar with the presentation [5].

The commonest locations of classic actinomycosis infection are cervicofacial, thoracic, abdominal, and pelvic. *A. israelii* and *A. gerencseriae* are the causative agents in 70% of infections [6]; however, *A. europaeus* is a much more rare species most often associated with skin and soft tissue infections [7, 8]. *A. europaeus* can display intrinsic resistance to many antibiotics including ciprofloxacin, erythromycin, and linezolid, as well as reduced susceptibility to piperacillin/tazobactam [9]. This is an additional difficulty in the treatment of *A. europaeus* and limits the nonpenicillin-based antimicrobial options.

Breast abscesses caused by *A. europaeus* are very rare but have been described in the literature. This case is novel in that the patient is penicillin allergic, therefore making suitable outpatient parenteral antibiotic therapy (OPAT) choices extremely difficult.

## 2. Case Report

A 69-year-old lady with a background of penicillin allergy and left-sided breast cancer treated with mastectomy and axillary node clearance was admitted to the hospital in Liverpool in 2016 with a right-sided breast abscess. The patient had been aware of a lump in her right breast for 3 years and had been told it was a sebaceous cyst. However, it had become painful, and she was now feeling unwell and tachycardic but remained apyrexial.

Antibiotic therapy was initiated with IV clindamycin 450 mg TDS and IV teicoplanin 12 mg/kg BD and then with 12 mg/kg OD after 2 days. The abscess was aspirated and subsequently incised and drained under local anaesthetic. The frank pus drained was malodorous, so oral metronidazole 500 mg TDS was added on. As there was no clinical improvement after 48 hours, a second incision and drainage was performed under general anaesthetic, revealing a large abscess cavity extending 10 cm into the right breast and 7 cm into the left mastectomy scar. This combined with continued triple antibiotic therapy resulted in clinical improvement, so after 4 days of IV therapy, antibiotics were stepped down to oral erythromycin 500 mg QDS of 7-day course and metronidazole 500 mg TDS of 10-day course and the patient was discharged. The abscess healed well in the community.

Twenty-one days after sending aspirated pus for MC + S, *Actinomyces europaeus* was grown in the culture. The patient was still clinically well, the abscess was healing well, and no underlying chest source of infection was identified.

The breast abscess pus was sent to the microbiology laboratory for testing. There were Gram-positive cocci visualised on the direct Gram stain, but there was no evidence of any Gram-positive bacilli. The pus was cultured onto the following plates: blood agar, MacConkey agar, selective anaerobic agar with a 5-microgram disc, and a fastidious anaerobic agar (FAA) plate in accordance with the laboratory standard operating procedure (SOP) based on the UK Standards for Microbiology Investigations (SMI) [10]. After 4 days of growth, two colonies grew on the direct FAA plate, which were different. The first colony was identified, using a matrix-assisted laser desorption/ionisation (MALDI-TOF) machine, as a *Staphylococcus epidermidis*. The MALDI-TOF equipment used was Bruker Microflex utilising MALDI Biotyper Version 3.1 software. The MS method of identification was the AutoX setting, and the software library was the BDAL and Filamentous Fungi Library Version 1.0. The second colony failed to be identified using MALDI. Therefore, the second organism was subbed onto an *Actinomyces* plate (blood agar with supplementary metronidazole and nalidoxic acid) in accordance with the local SOP. Four days later, there was fine growth on the *Actinomyces* plate. Sensitivities were obtained via the British Society for Antimicrobial Chemotherapy (BSAC) method of disc diffusion. The organism was sensitive to penicillin 42 mm (sensitivity > 23 mm), erythromycin 40 mm (sensitivity > 10 mm), vancomycin 30 mm (no breakpoint available), tetracycline 42 mm (no breakpoint available), and ciprofloxacin 22 mm (no breakpoint available). The organism was initially identified as *Actinomyces europaeus*, but the MALDI score was only 1.82; therefore, an extraction MALDI was performed to increase the MALDI score, so a more confident identification of the organism was obtained. The subsequent MALDI identity was *Actinomyces europaeus* with a score of 2.10. As per the guidance from the SMI, the organism was sloped and sent to the Anaerobic Reference Unit at Colindale. The ID was confirmed as *Actinomyces europaeus* by partial sequencing of 16sRNA.

Microbiological advice consisted of a long course of antibiotics to eradicate the infection, and the first-line treatment was 2 weeks of IV ceftriaxone followed by 6 months of oral amoxicillin. This was not feasible due to the patient's penicillin allergy.

Second-line advice was 2 weeks of IV erythromycin followed by 6 months of oral erythromycin. However, this was also not feasible. The outpatient parenteral antibiotic therapy (OPAT) service enables patients to receive IV therapy in the community but was unable to provide the four times daily dosing required for IV erythromycin. The OPAT service was only able to provide twice daily regimens.

The third-line approach was acceptable: 2 weeks of IV tigecycline given in twice daily doses followed by 6 months of oral doxycycline. The patient had a midline inserted and received her IV therapy in the community with OPAT services.

After completing the IV tigecycline course with no issues, the patient complained of side effects following 6 weeks of oral doxycycline therapy: oral thrush and leg blisters. Antibiotic therapy was switched to oral clarithromycin to complete a 6-month course of oral antibiotics. There has been no evidence of recurrence to date.

## 3. Discussion

This case is novel because the patient involved is allergic to penicillin. The case was further complicated by the need to design a parenteral antimicrobial regimen that was logistically feasible to deliver on an outpatient basis.

The *Actinomyces* species are generally sensitive to penicillin, so this is recommended as the first-line therapy. Consideration may also be given to concomitant bacteria that may be resistant to penicillins and continue the inflammation after the actinomycetes have been treated; for example, dual therapy may be utilised with metronidazole for anaerobic cover in addition to penicillin [11]. Prolonged antibiotic courses are required (typically 6 months) in order for the treatment to penetrate abscesses and infected tissue [6]. However, combined surgical-medical treatment such as resection of infected tissue followed by antimicrobial therapy may shorten the required antimicrobial therapy duration [6]^.^ Therapy duration of less than 3 months is not recommended due to the high risk of recurrence [12]. Treatment of all forms of actinomycosis traditionally involves high-dose IV penicillin G for two to six weeks of duration, followed by a step down to oral penicillin V for 6–12 months [3, 13]. Prolonged courses of parenteral antibiotics can be challenging to provide as they require specialist administration. Outpatient parenteral antibiotic therapy (OPAT) services can facilitate this.

OPAT was initially developed in the US in the 1970s for the management of children with cystic fibrosis [14], and it has since been extrapolated to treat many conditions worldwide. Outpatient therapy avoids the costs associated with inpatient admission and freeing up more inpatient beds while reducing the risk of hospital-associated infections [15] and improving the quality of life by allowing the patient to remain at home.

Care must be taken to ensure that the patient is suitable for outpatient management as the condition must be stable with low risk of deterioration [16]. The patient in question fulfilled this criterion because she had been managing her condition in the community for 2 weeks prior to being recalled to hospital and she had a good family support network to help her if required.

OPAT services can be delivered in 3 ways: at an outpatient clinic, by a health care professional at the patient's home, or self-administration by the patient. Self-administration enables more flexibility in dose frequency; however, the patient in question was frail with comorbidities that made this option difficult. Funding and capacity of OPAT services exclude the ability to provide therapies that require frequent dosing either by staff at home or in a clinic—this factor rendered erythromycin inappropriate as an outpatient therapy option.

There is much conflict in the literature regarding *A. europaeus* sensitivities and resistance. *A. europaeus* is widely reported as the species of *Actinomyces* that has the highest resistance rates [9, 17, 18], and some in vitro studies have described evidence of resistance or reduced susceptibility of *A. europaeus* to erythromycin [9, 17], whereas others have described erythromycin to be the most active antimicrobial agent in the treatment of *A. europaeus* [9]. Good sensitivity to erythromycin was demonstrated in this isolate. This could be the impact of evolving antibiotic resistance secondary to antimicrobial misuse and overuse, and especially macrolide resistance is being increasingly reported in skin and soft tissue infections [19]. However, erythromycin is recommended as a nonpenicillin antibiotic therapy for treatment of *Actinomyces* infection [3, 20], and tetracyclines have also been recommended [9]. The use of tetracyclines highlights the importance of correctly identifying the *Actinomyces* species as *A. europaeus* is generally sensitive, while other species display resistance to tetracyclines [20].

## 4. Conclusion

This report considers the difficulty in identifying a clinically viable antimicrobial agent for the treatment of *A. europaeus* that is also logistically feasible as an outpatient parenteral therapy. This concern may become more prevalent as the push in NHS management is towards care based in the community rather than hospitals. This will help to reduce the hospital-associated morbidity such as infections and ease the pressures on bed availability.
